# Transcription factors *Tp73*, *Cebpd*, *Pax6,* and *Spi1* rather than DNA methylation regulate chronic transcriptomics changes after experimental traumatic brain injury

**DOI:** 10.1186/s40478-018-0519-z

**Published:** 2018-02-27

**Authors:** Anssi Lipponen, Assam El-Osta, Antony Kaspi, Mark Ziemann, Ishant Khurana, Harikrishnan KN, Vicente Navarro-Ferrandis, Noora Puhakka, Jussi Paananen, Asla Pitkänen

**Affiliations:** 1 Epilepsy Research Laboratory, A. I. Virtanen Institute for Molecular Sciences, University of Eastern Finland, PO Box 1627, FIN-70211 Kuopio, Finland; 2 Epigenetics in Human Health and Disease Laboratory, Central Clinical School, Faculty of Medicine, Monash University, Melbourne, VIC Australia; 3 Prince of Wales Hospital, The Chinese University of Hong Kong, Sha Tin, Hong Kong SAR; 4 Institute of Biomedicine, University of Eastern Finland, Kuopio, Finland; 5 University of Eastern Finland Bioinformatics Center, University of Eastern Finland, Kuopio, Finland

**Keywords:** DNA methylation, LINCS analysis, MBD-seq, Recovery, RNA-seq, Treatment

## Abstract

Traumatic brain injury (TBI) induces a wide variety of cellular and molecular changes that can continue for days to weeks to months, leading to functional impairments. Currently, there are no pharmacotherapies in clinical use that favorably modify the post-TBI outcome, due in part to limited understanding of the mechanisms of TBI-induced pathologies. Our system biology analysis tested the hypothesis that chronic transcriptomics changes induced by TBI are controlled by altered DNA-methylation in gene promoter areas or by transcription factors. We performed genome-wide methyl binding domain (MBD)-sequencing (seq) and RNA-seq in perilesional, thalamic, and hippocampal tissue sampled at 3 months after TBI induced by lateral fluid percussion in adult male Sprague-Dawley rats. We investigated the regulated molecular networks and mechanisms underlying the chronic regulation, particularly DNA methylation and transcription factors. Finally, we identified compounds that modulate the transcriptomics changes and could be repurposed to improve recovery. Unexpectedly, DNA methylation was not a major regulator of chronic post-TBI transcriptomics changes. On the other hand, the transcription factors ***Cebpd***, ***Pax6***, ***Spi1,*** and ***Tp73*** were upregulated at 3 months after TBI (False discovery rate < 0.05), which was validated using digital droplet polymerase chain reaction. Transcription regulatory network analysis revealed that these transcription factors regulate apoptosis, inflammation, and microglia, which are well-known contributors to secondary damage after TBI. Library of Integrated Network-based Cellular Signatures (LINCS) analysis identified 118 pharmacotherapies that regulate the expression of ***Cebpd***, ***Pax6***, ***Spi1,*** and ***Tp73***. Of these, the antidepressant and/or antipsychotic compounds trimipramine, rolipramine, fluspirilene, and chlorpromazine, as well as the anti-cancer therapies pimasertib, tamoxifen, and vorinostat were strong regulators of the identified transcription factors, suggesting their potential to modulate the regulated transcriptomics networks to improve post-TBI recovery.

**Electronic supplementary material:**

The online version of this article (10.1186/s40478-018-0519-z) contains supplementary material, which is available to authorized users.

## Introduction

Every year, 2.5 million people in Europe and the USA sustain traumatic brain injury (TBI) [15, 28, 74]. TBI is a major cause of disability and death in patients younger than 45 years of age [65]. Despite a large number of preclinical and clinical studies, an effective pharmacotherapy to improve post-TBI outcome is still lacking [22, 25, 76]. This is due in part to the complexity of the secondary pathologies induced by TBI, including neurodegeneration, inflammation, oxidative stress, axonal and myelin injury, and vascular changes [64, 79, 80]. These pathologies progress in parallel and serial time windows over weeks to months in experimental models [7, 46, 91, 112] and humans [36, 49, 98]. As these observations suggest complex and long-lasting transcriptomics regulation, we propose that a network therapy rather than a monotherapy approach will be more effective for repair of the ongoing damage.

To date, a number of studies have investigated transcriptomics changes at a genome-wide scale at 24-48 h after TBI [14, 17, 38, 42, 47, 48, 59, 63, 78, 85, 97, 99, 104, 122, 123, 125, 128–130, 136]. Of these 19 genome-wide gene expression studies, 11 report dysregulation of transcription factors in the acute post-TBI phase [17, 48, 59, 78, 85, 97, 104, 123, 128–130]. Four hypothesis-driven analyses focusing on individual transcription factors report the dysregulation of *Jun* [133], *Cebpd* [105], *Runx1* [70], and *Olig2* [9] in the acute post-TBI phase. Most of the studies, however, analyzed only acute post-TBI time-points and a single brain area, typically the hippocampus or cortex. Further, very few studies have explored the mechanisms that regulate post-TBI gene expression, such as genome-wide DNA methylation [20, 39, 83].

We hypothesized that TBI results in chronic transcriptomics changes that are controlled by DNA-methylation changes in the gene promoter areas or by transcription factors. To test this, we induced TBI in rats by lateral fluid-percussion, and subjected the perilesional cortex, ipsilateral thalamus, and ipsilateral hippocampus to MBD-seq and RNA-seq. As bioinformatics analysis and laboratory validation indicated that transcription factors rather than DNA methylation regulate chronic transcriptomics changes, we further conducted LINCS analysis to identify compounds that regulate gene expression of these transcription factors and could therefore be repurposed to improve post-TBI outcome via transcription factor-mediated mechanisms.

## Materials and methods

### Animals

TBI was induced by lateral fluid-percussion injury (FPI) with an impact pressure of 3.30 ± 0.01 atm in 14 adult male Sprague-Dawley rats (330–370 g at the time of TBI or sham operation; Harlan, The Netherlands) as previously described [54, 81]. Eleven sham-operated animals served as experimental controls. At 3 months after TBI, the perilesional cortex, thalamus, and hippocampus were collected as described in Lipponen et al. [69]. Briefly, the rats were anesthetized with 5% isoflurane and decapitated. The brain was removed from the skull, flushed with 0.9% cold (4 °C) sodium chloride, and placed onto a slicing matrix on ice (#15007, Rodent Brain Matrix, Ted Pella, Inc., Redding, CA, USA). Two 2-mm-thick coronal slices were cut (between − 2.2 and − 6.2 from the bregma), from which the perilesional cortex, ipsilateral thalamus, and ipsilateral hippocampus (including dentate gyrus) were dissected on top of the light table under the magnifying glass. Brain tissue samples were snap-frozen in liquid nitrogen, and stored at -70 °C until RNA and DNA extraction.

All animal operations were approved by The Animal Ethics Committee of the Provincial Government of Southern Finland and carried out according to the guidelines of the European Community Council Directives 2010/63/EU.

### Preparation of MBD- and RNA-seq libraries and sequencing

#### DNA and RNA extraction

Brain tissue from five TBI and five sham-operated rats was used for methyl-binding domain sequencing (MBD-seq) and RNA-sequencing (RNA-seq). DNA and RNA were co-purified from the perilesional cortex, ipsilateral hippocampus, or ipsilateral thalamus using a DNeasy Blood&Tissue kit (#69504, Qiagen, Hilden, Germany). Quality control of the total RNA was performed using a MultiNA electrophoresis device (Shimazu, Kyoto, Japan).

#### RNA-seq library and sequencing

The mRNA library preparation and RNA-sequencing were performed as described in Lipponen et al. [69]. Briefly, mRNA was enriched using Dynabeads Oligo (dT)25 beads (#61002, Invitrogen, Carlsbad, CA, USA), and the sequencing libraries were compiled with the NEBNext mRNA Library Prep Reagent Set (#E6100S, New England Biolabs, Ipswich, MA, USA). Quality control of the sequencing libraries was performed with a MultiNA electrophoresis device (Shimazu, Kyoto, Japan). Sequencing of the mRNA libraries for the perilesional cortex and hippocampus was carried out with an Illumina Genome Analyzer IIx (San Diego, CA, USA), and for the thalamus using an Illumina HiSeq 2000 (San Diego, CA, USA). The Illumina Off-Line Basecaller v1.8 was used for base-calling. RNA-seq raw data can be downloaded from the NCBI Gene Expression Omnibus (GEO; series accession number GSE80174).

#### MBD-seq library and sequencing

For MBD-seq, 2 μg of DNA was fragmented by sonication, and the quality was controlled with a MultiNA electrophoresis device (Shimazu, Kyoto, Japan). Methylated DNA was enriched with a 2-M sodium chloride elution using MethylMiner™kit (Thermo Fischer Scientific, Waltham, MA, USA), and quantified using a Qubit fluorometer (Thermo FisherScientific, Waltham, MA, USA). The sequencing library was prepared from 5 ng of enriched methylated DNA using an NEB Next DNA library kit (#E6040S, New England Biolabs, Ipswich, MA, USA). Then, MBD-sequencing and base-calling for the perilesional cortex, hippocampus, and thalamus were carried out as described above. Raw MBD-seq data was saved to the NCBI Gene Expression Omnibus (GEO; series accession number GSE107837).

### Mapping of sequencing data, and identification of differentially methylated regions and differentially expressing genes

#### Methylation

Quality control of the MBD-sequencing raw reads was performed using FastQC [3]. Sequencing raw reads were mapped to the Ensemble RN5 genome with Spliced Transcripts Alignment to a Reference (STAR) software (version 2.3.0e_r291) [24] with parameter alignIntronMax 1 to prohibit splicing and allow genomic mapping. The mapping percentages were 71.2 ± 4.0% for the perilesional cortex, 63.3 ± 3.2% for the ipsilateral hippocampus, and 71.3 ± 2.7% for the ipsilateral thalamus. Differentially-methylated gene promoters (5000 bp upstream and 200 bp downstream from the transcription start site), exons, and gene body area were identified with blocksStats function in the Repitools 1.21.1 R package [115] with R version 3.1.0. The adjusted *p*-value was calculated with a Benjamini–Hochberg false discovery rate (FDR). DNA methylation was considered significantly changed if the FDR was < 0.05.

#### Gene expression

RNA-seq quality control, mapping, and identification of differentially expressed genes were previously described in detail (Lipponen et al. 2016) [69]. Shortly, quality control of the RNA-seq reads was performed using FastQC [3] and reads were aligned to the Ensemble RN5 genome with STAR software (version 2.3.0e_r291) [24]. Differentially expressed genes were identified with DEseq2 [72] R package (R version 3.1.0) and the Benjamini–Hochberg false discovery rate (FDR) was used to calculate the adjusted *p*-value. Gene expression was considered to be significantly differentially expressed when FDR < 0.05.

### Effect of DNA methylation in the promoter, exon, or gene body region on gene expression

#### Gene set enrichment analysis

To analyze the effect of DNA methylation located in the gene promoter, exon, or gene body areas on gene expression, we performed Gene Set Enrichment Analysis (GSEA) [116]. First, we prepared ranked lists from the gene expression data in the perilesional cortex, hippocampus, and thalamus by ranking the genes in order according to the p-value of mRNA differential expression. Upregulated genes were assigned with a positive rank number and downregulated genes with a negative rank number. Then, we generated three gene sets (genes with differentially methylated promoters, exons, or gene body areas) from each of the three brain areas (perilesional cortex, hippocampus, thalamus). Enrichment of these sets within the ranked lists was studied using GSEA, and enrichment was considered significant when the FDR q-value was < 0.05.

#### Linear regression analysis

To confirm the GSEA results, we analyzed the association of DNA methylation in the gene promoter, exon, and gene body areas on gene expression using two different linear regression models: (a) association of DNA methylation on gene expression, (b) association of TBI on gene expression via DNA methylation. Regression analysis was carried out with lm-function in R v3.1.0. Genes with average mRNA read number < 50 were filtered out from the analysis. Regression was considered significant when FDR < 0.05.

### Validation of gene promoter methylation and gene expression

Digital droplet polymerase chain reaction (ddPCR) and pyrosequencing were used to confirm the gene expression changes in the RNA-seq and the methylation changes in the MBD-seq, respectively, of the four top hits. Of the four top hits, ***Gpr12*** and ***Lrp1b*** were downregulated in the mRNA-seq and showed increased promoter methylation in the MBD-seq in the perilesional cortex, ***Ppid*** showed increased promoter methylation in the thalamus, and ***Wdr26*** showed increased promoter methylation in the hippocampus (Table 1).

**Table 1:** Gene promoter methylation and gene expression of *Wdr26, Lrp1b, Ppid* and *Gpr12* in perilesional cortex, hippocampus and ipsilateral thalamus according MBD and RNA-seq

		*Wdr26*	*Lrp1b*	*Ppid*	*Gpr12*
Perilesional Cx	***Gene expression***				
	log2FC	− 0.161	**− 0.441**	− 0.222	**− 0.531**
	FDR	0.300607	**0.022273**	0.242403	**0.000197**
	***Methylation***				
	log2FC	0.313	**1.402**	0.038	**1.407**
	FDR	1	**0.01901**	1	**0.014662**
Hippocampus	***Gene expression***				
	log2FC	−0.129	−0.166	−0.036	0.224
	FDR	0.999825	0.999825	0.999825	0.999825
	***Methylation***				
	log2FC	**2.660**	−0.602	**1.930**	0.179
	FDR	**1.87E-14**	1	**1.4E-07**	1
Thalamus	***Gene expression***				
	log2FC	0.140	−0.097	−0.074	**− 0.687**
	FDR	0.721246	0.795279	0.873954	**0.000388**
	***Methylation***				
	log2FC	0.241	0.159	0.059	−0.014
	FDR	1	1	1	1

Abbreviations: *Cx* perilesional cortex, *FC* fold-change. ***Statistical significances:*** log2FC change and corresponding FDR (< 0.05) are shown in bolded font

#### Extraction of RNA for ddPCR and DNA for pyrosequencing

Brain tissue from nine TBI and six sham-operated animals was collected as described above. The animals belonged to the same cohort of injured rats used for the RNA-seq and MBD-seq analyses.

RNA and DNA were extracted simultaneously from the perilesional cortex, thalamus, or hippocampus using a *mir*Vana miRNA isolation kit (#AM1560, Life Technologies (Ambion) Carlsbad, CA, USA), QIAshredder (#79654, Qiagen), and AllPrep DNA/RNA Mini Kit (#80204, Qiagen) as previously described [90]. Briefly, to avoid clogging the spin columns, brain tissue was divided into 2–5 pieces (each ~ 10 mg) on dry ice. Each tissue piece was then placed into a 2-ml microcentrifuge tube together with one metal ball and 800 μl of Ambion Lysis/binding buffer, and homogenized with a TissueLyser (Qiagen) for 3 min (30 Hz). For further homogenization, the lysate was transferred to a QIAshedder spin column and centrifuged (16,000 g) for 2 min at 4 °C. Flow-through lysate was transferred back to the QIAshedder spin column and centrifuged again. For DNA extraction, lysate was transferred to a Qiagen All Prep DNA spin column and centrifuged (10,000 g) for 1 min at room temperature. The spin-column was washed and eluted according to the instructions provided in the AllPrep DNA/RNA Mini Kit for DNA extraction.

Flow-through from the All Prep DNA spin column was used for RNA extraction using a *mir*Vana miRNA isolation kit. Briefly, miRNA homogenate additive (70 μl) was added to the flow-through. The mixture was vigorously vortexed for 30 s and then incubated on ice for 10 min. Acid-phenol:chloroform (700 μl) was then added, mixed, and centrifuged (16,000 g) for 30 s. The aqueous upper phase was transferred to a new microcentrifuge tube. Five hundred microliters of water was added to the lower phase, mixed, and centrifuged (16,000 g) for 30 s. The upper aqueous phase was collected into the same tube as the aqueous phase from the previous extraction cycle. Then, 100% ethanol (625 μl) was added to the tube, mixed, and transferred to the *mir*Vana miRNA isolation spin column. Finally, RNA was washed and eluted from the spin column according to instructions provided with the *mir*Vana miRNA isolation kit. Finally, RNA extracted from each brain region was pooled.

#### Pyrosequencing

Percentage (ratio of methylated/nonmethylated DNA _*_ 100) of DNA methylated cytosines at a given CpG site in the ***Ppid****,* ***Lrp1b****, or* ***Wrd26*** gene promoters, or at two CpG sites in the ***Gpr12*** promoter was measured with pyrosequencing using the PSQ 96MA 2.1 platform (Biotage AB, Uppsala, Sweden) in the Genome Center of the University of London (Additional file 1). The Mann-Whitney U test was used to assess the significance of the difference in the percentage of methylation between the TBI and sham-operated animals (*p* < 0.05 was considered statistically significant) (Table 2).

**Table 2:** Validation of gene promoter methylation by pyrosequencing and gene expression by ddPCR in the perilesional cortex, thalamus and hippocampus. As negative controls, we also assessed the methylation and gene expression in the ipsilateral hippocampus and thalamus. *Wdr26, Lrp1b,* and *Ppid* genes had only one CpG site whereas *Gpr12* gene had two CpG sites

		*Wdr26*	*Lrp1b*	*Ppid*	*Gpr12*	
Perilesional Cx	***Gene expression***					
	Log2FC	**−0.94944**	− 0.17498	**− 0.60015**	−0.37159	
	p-value	**0.007592**	0.7756	**0.03596**	0.3277	
	***Methylation***					
	Average methylation (%)	85.31	67.28	59.52	CpG site1: 73.43	CpG site2: 76.57
	Difference TBI *-* sham (%)	1.07	−0.17	2.39	CpG site1:0.43	CpG site2:0.35
	p-value	0.1135	0.9546	0.3636	CpG site1: 0.8639	CpG site2: 1
Hippocampus	***Gene expression***					
	Log2FC	−0.6746	−0.6457	−1.3397	−0.3415	
	p-value	0.366	0.1375	0.366	0.366	
	***Methylation***					
	Average methylation (%)	87.22	59.92	57.25	CpG site1: 69.06	CpG site2: 73.48
	Difference TBI *-* sham (%)	1.10	−2.73	2.65	CpG site1: 1.70	CpG site2: −0.59
	p-value	0.366	0.2343	0.366	CpG site1: 0.5338	CpG site2: 1
Thalamus	***Gene expression***					
	Log2FC	−0.8936	−0.4964	−0.9309	**−1.3735**	
	p-value	0.181	0.3277	0.06633	**0.001598**	
	***Methylation***					
	Average methylation (%)	85.11	65.82	49.95	CpG site1: 74.23	CpG site2: 77.40
	Difference TBI *-* sham (%)	1.07	−2.33	7.69	CpG site1: −0.95	CpG site2: −0.93
	p-value	0.1469	0.5287	0.3884	CpG site1: 0.7756	CpG site2: 0.1135

*Abbreviations: Cx* cortex, *FC* fold-change. ***Statistical significances:*** log2FC change and corresponding p-value (< 0.05) are shown in bolded font

#### ddPCR

RNA quality was checked with a 2100 Bioanalyzer (Agilent, Santa Clara, CA, USA) and RNA 6000 Nano Kit (#5067–1511, Agilent). The RNA integrity number (RIN) was > 8.0 in all but one sample (RIN 7.8). Therefore, 50 ng of RNA from all 15 samples was translated to cDNA using an iScript Advanced cDNA synthesis kit (#172–5038, BioRad, Hercules, CA, USA). Gene expression of ***Ppid,*** ***Gpr12,*** ***Lrp1b,*** *and* ***Wrd26*** was validated by ddPCR using ***Actb*** as a reference gene. ddPCR was conducted using QX200 ddPCR EvaGreen Supermix solution (#186–4033, BioRad) and a PrimerPCR gene expression assay (Actb; #dRnoEG5146006, Gpr12; #dRnoEG5125945, Lrp1b; #dRnoEG5140109, Ppid; #dRnoEG5125166, Wdr26; #dRnoEG5145659, BioRad) according to the manufacturer’s instructions. Droplets were generated and their fluorescence measured with a QX200 Droplet Digital PCR System (BioRad). Fluorescent droplets were classified as “positive” and “negative”, and then the concentration of each gene (copy/μl) was calculated using QuantaSoft 1.7.4 (BioRad). Target gene concentrations were normalized according to the ***Actb*** reference gene, log2 fold-changes were calculated, and the significance in the difference in droplet counts between the TBI and sham-operated rats was assessed using the Mann-Whitney U test (Table 2).

### Identification of transcription factors that chronically regulate post-TBI transcriptomics

#### Transcription regulatory network of the perilesional cortex and ipsilateral thalamus

To analyze the regulation of post-TBI transcriptomics by transcription factors in the perilesional cortex and ipsilateral thalamus, and to further visualize their target genes, we downloaded the transcription regulatory network (TRN) from the SignaLink 2.0 database [31, 60]. Then, RNA-seq data from both brain areas (fold-change and FDR between sham-operated experimental controls and TBI animals) was integrated with the SignaLink 2.0 TRN in Cytoscape 3.4 [108]. To visualize the regulated targets of each selected transcription factor, differentially expressed transcription factors in mRNA-seq (FDR < 0.05 and log2FC < − 1 or > 1) and their nearest downstream neighbors in the TRN were detached from the SignaLink 2.0 network. Further analysis focused on the four transcription factors that had the highest number of regulated target genes in TRN.

### Validation of gene expression of the top four transcription factors

#### ddPCR

Digital droplet PCR of the four top transcription factors (***Cebpd, Pax6***, ***Spi1***, and ***Tp73***) using ***Actb*** as a reference gene was performed to confirm the change in the expression of transcription factors coding genes. Analysis was performed using the same RNA samples, cDNA synthesis method, and ddPCR EvaGreen Supermix solution as for the validation of MBD-seq data (see above). A PrimerPCR gene expression assay (Actb; #dRnoEG5146006, Cebpd; #dRnoEG5126239, Pax6; #dRnoEG5125771, Spi1; #dRnoEG5139059, Tp73; #dRnoEG5139860, BioRad) was performed according to the manufacturer’s instructions. Droplets were generated and their fluorescence measured with the QX200 Droplet Digital PCR System. QuantaSoft 1.7.4 was used to classify droplets as “positive” and “negative”, and to calculate the concentration of gene copies/μl as described earlier. Finally, the target gene concentration was normalized with the reference gene concentration, the log2 fold-change of the concentration was calculated, and the significance in the difference in concentrations between the TBI and sham-operated rats was assessed using the Mann-Whitney U test.

### Identification of compounds modifying the transcription factor gene expression with the LINCS database

#### Compounds modifying transcription factors gene expression

Next, we searched the LINCS database (http://data.lincscloud.org.s3.amazonaws.com/index.html) to identify compounds that modify the expression of the four top transcription factors revealed by the SignaLink 2.0 database analysis. An in-house–created R script was used to run LINCS through an application programming interface. Reproducible compound-induced transcriptomics changes in terminally differentiated neurons (NEU), terminally differentiated neurons treated with KCl (NEU.KCL), and induced pluripotent stem cells-derived neural progenitor cells (NPC) were included in the analysis. As a result, we obtained a list of compounds that modulated the expression of transcription factors in one to three cell lines (i.e.*,* transcription factors were within the 100 most upregulated or 100 most downregulated genes by a given compound).

## Results

### TBI-induced gene expression changes after TBI were most prominent in the perilesional cortex and were associated with DNA methylation at the gene promoter region

#### GSEA analysis of MBD-seq data

**Fig. 1:**
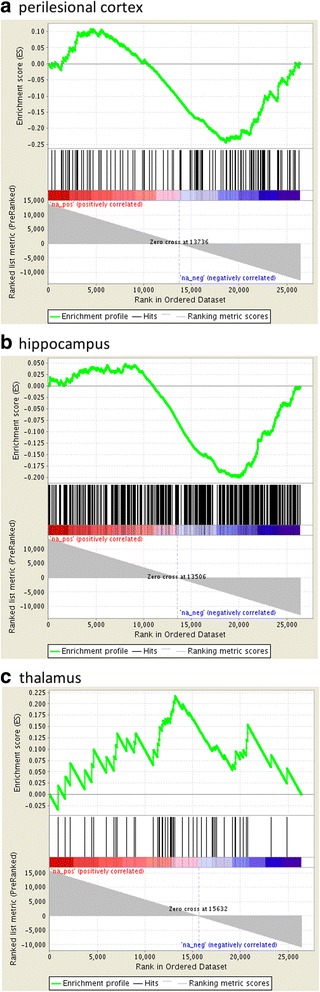
Enrichment scores of Gene Set Enrichment Analysis (GSEA) of DNA methylation in gene promoters in the (**a**) perilesional cortex (**b**) ipsilateral hippocampus, and (**c**) ipsilateral thalamus at 3 months after TBI. GSEA indicated significant negative enrichment in the perilesional cortex (FDR q-val. 0.046), but not in the ipsilateral hippocampus (FDR q-val. 0.079) or ipsilateral thalamus (FDR q-val. 0.828)

GSEA analysis of the perilesional cortex MBD-seq data suggested that altered DNA methylation in the gene promoter area was inversely associated with the global gene expression profile (FDR q-val. 0.046) (Fig. 1). Altered DNA methylation in the gene promoter area in the hippocampal (FDR q-val. 0.079) or thalamic samples (FDR q-val. 0.828), however, was not associated with regulated gene expression. Interestingly, changes in DNA methylation in exons were not associated with gene expression in the perilesional cortex (FDR q-val. 0.243), hippocampus (FDR q-val. 0.464), or thalamus (FDR q-val. 0.372). DNA methylation in the gene body area also was not associated with transcriptomics changes in the perilesional cortex (FDR q-val. 0.083), thalamus (FDR q-val. 0.256), or hippocampus (FDR q-val. 0.631).

#### Linear regression analysis of the MBD-seq data

To find individual genes affected by DNA methylation and TBI from the global DNA methylation profile, we performed a regression analysis to separately assess the TBI effect and methylation effect on gene expression. In the perilesional cortex and ipsilateral thalamus, however, we observed no TBI-induced effect on DNA methylation of individual genes when the gene promoter, exon, or gene body areas were analyzed separately. In contrast, in the hippocampus, TBI affected the DNA methylation of ***Crybg3*** (FDR = 0.0347, estimate = − 1.105 (95% confidence interval [CI]: − 1.2517 to − 0.9598) and ***Mak16*** (FDR = 0.0356, estimate = 4.596, 95% CI: 3.9705 to 5.2219) promoters, which affected their target gene expression. in the hippocampus, no TBI effect on methylation was detected in the exon and gene body regions.

We also used regression analysis to investigate the DNA methylation effect on gene expression. DNA methylation in any of the genomic areas did not appear to affect gene expression in any of the studied brain regions.

#### Methylation of the promoter area

Analysis of MBD-seq data revealed significantly regulated DNA methylation in the promoter regions of 29 genes (all with increased methylation) in the perilesional cortex (FDR < 0.05). In four of 29 genes (***RGD1566265***, ***Nap1l2, Lrp1b,*** and ***Gpr12****)*, an increase in promoter methylation was associated with reduced gene expression in the corresponding perilesional cortex (FDR < 0.05). In the hippocampus, none of the 166 methylation changes (97 increased and 69 decreased) in the promoter area were associated with changes in gene expression. In the thalamus, no alterations in promoter methylation were found (FDR < 0.05).

#### Methylation of exons

Methylation in exons of 20 genes (15 increased and 5 decreased) was regulated in the hippocampus, but none of the methylation changes were associated with altered expression of the corresponding gene in the RNA-seq data (FDR < 0.05). In the perilesional cortex and thalamus, we observed no changes in exon methylation (FDR < 0.05).

#### Methylation of gene body areas

Methylation of the gene body area was not changed in any of the brain areas studied (FDR < 0.05).

### Validation of MBD-seq using pyrosequencing and RNA-seq data using ddPCR failed to confirm a link between promoter methylation and changed gene expression in the perilesional cortex at 3 months after TBI

#### Validation of gene promoter area methylation and gene expression

Promoter methylation and expression of ***Lrp1b***, ***Gpr12, Wrd26,*** and ***Ppid*** genes in the perilesional cortex were validated with pyrosequencing and ddPCR, respectively, in animals from the same cohort used for the RNA-seq and MBD-seq studies (Table 2). The percentage of methylation per CpG site in the promoter region varied from 49.95 to 87.22%, depending on the gene or the methylation site in each gene (Table 2). Unexpectedly, pyrosequencing indicated that none of the four tested genes in the perilesional cortex had altered methylation in the gene promoter area (Table 2). ddPCR, however, confirmed reduced expression of ***Wrd26*** and ***Ppid*** in the perilesional cortex (Table 2).

### Post-TBI perilesional cortex showed a substantial increase in the expression of four transcription factors

#### Transcription factors regulating post-TBI gene expression

As we observed few methylation changes in relation to the large number of transcriptomic changes in the perilesional cortex and ipsilateral thalamus, we next assessed whether chronic post-TBI regulation of gene expression in these brain areas was controlled by transcription factors. To assess whether the transcriptomic changes observed in RNA-seq correspond to alterations in local cell populations we correlated the read counts of transcription factors with the read counts of neuronal, microglial and astroglial markers (Additional files 2 and 3).

#### Transcription regulatory network of the perilesional cortex

**Fig. 2:**
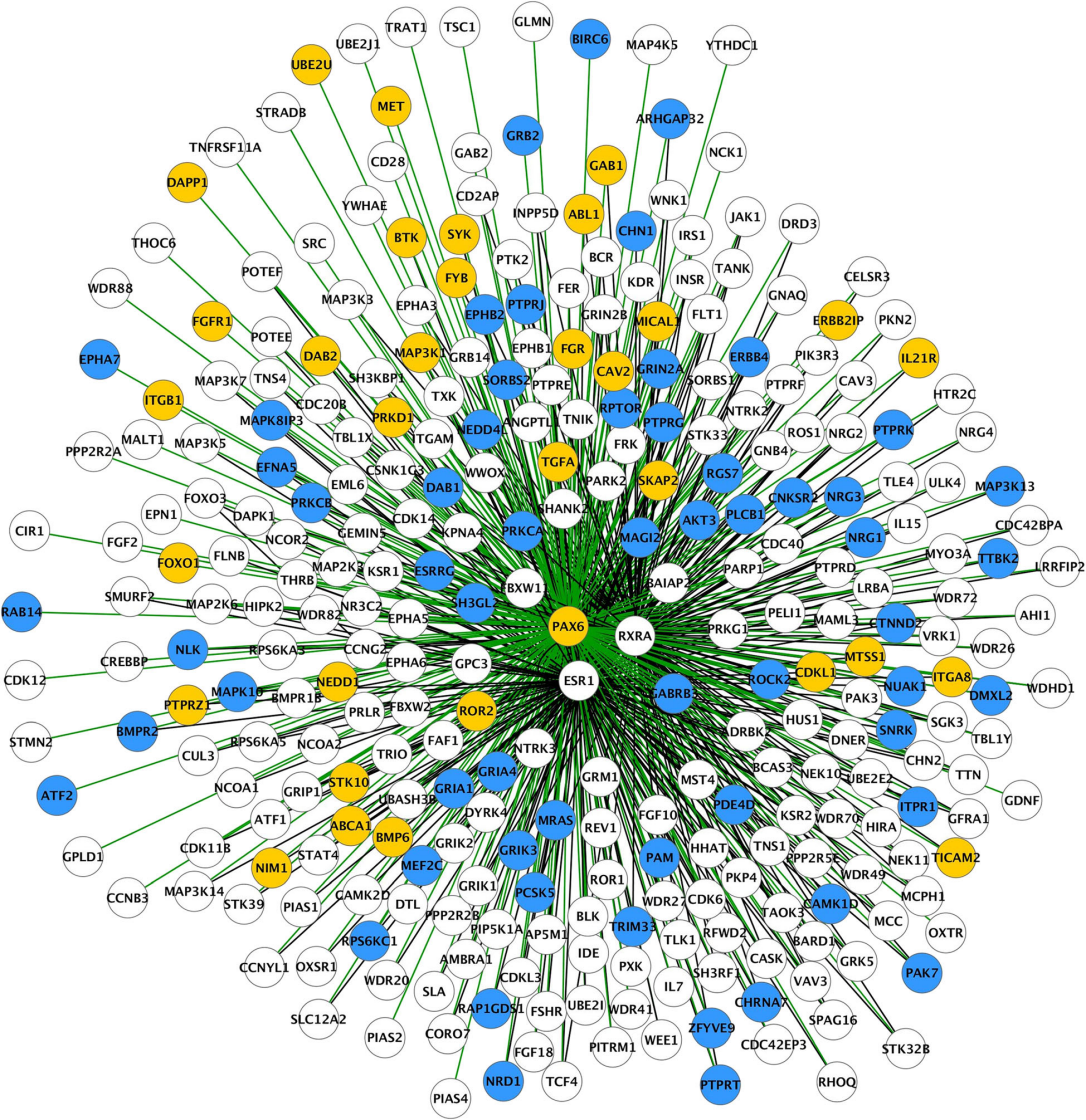
SignaLink transcription regulatory network (TRN) of *Pax6* and its targets in the perilesional cortex. SignaLink network analysis revealed 300 targets, of which 36 were upregulated and 59 were downregulated in the perilesional cortex 3 months post-TBI. Color codes: blue circle, downregulation after TBI; yellow circle, upregulation after TBI; white circle, no change in gene expression after TBI; green line, transcriptional interaction with *Pax6*; black line, inter-target interaction

**Fig. 3:**
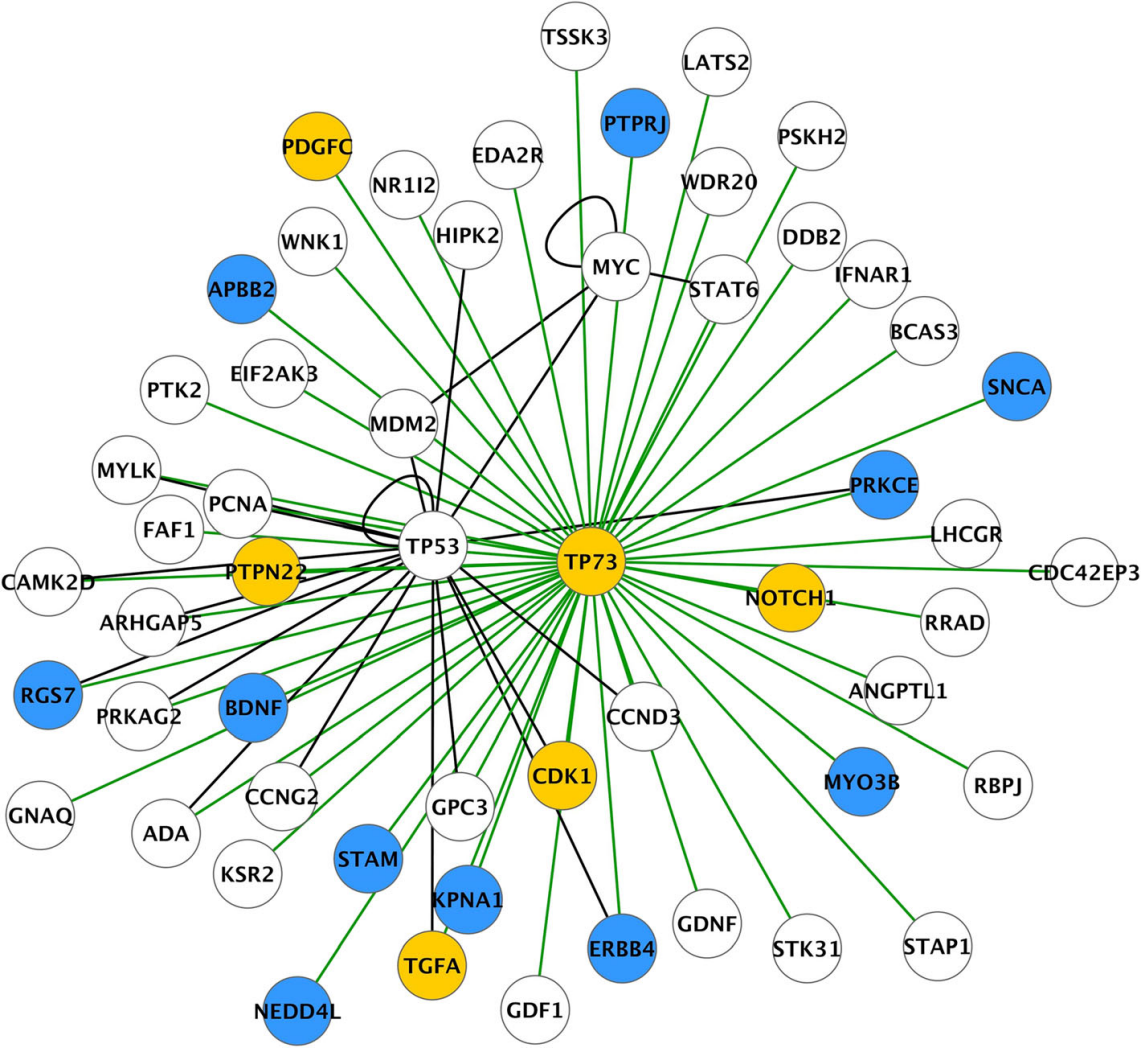
SignaLink transcription regulatory network (TRN) of *Tp73* and its targets in the perilesional cortex. SignaLink network analysis revealed 54 targets, of which five were upregulated and 11 were downregulated in the perilesional cortex 3 months post-TBI. Color codes: blue circle, downregulation after TBI; yellow circle, upregulation after TBI; white circle, no change in gene expression after TBI; green line, transcriptional interaction with *Tp73;* black line, inter-target interaction

In the perilesional cortex, integration of SignaLink 2.0 TRN and RNA-seq revealed increased expression in five transcription factors, ***Pax6*** (Fig. 2)***, Tp73*** (Fig. 3)*,* ***Cebpd***, ***Spi1***, and ***Myb***, and decreased expression in ***Etv4*** at 3 months post-TBI (Table 3)*.* ***Pax6*** had 300 targets in the TRN analysis, of which 32 were upregulated and 59 downregulated. ***Tp73*** had 54 targets in the TRN analysis, of which five were upregulated and 11 downregulated. ***Igf1,*** the only target of ***Cebpd***, was upregulated in the TRN analysis. ***Spi1*** had three targets in the TRN analysis, of which only ***Lsp1*** was upregulated. According to the TRN analysis, ***Etv4*** had one target, which did not show altered gene expression. In the TRN analysis, ***Myb*** had three targets, none of which showed altered expression in the perilesional cortex.

**Table 3:** SignaLink 2.0 transcription regulatory network (TRN) analysis of differentially expressed transcription factors and their up-regulated and down-regulated target genes in the perilesional cortex and the ipsilateral thalamus at 3 months after traumatic brain injury. The same targets that were up-regulated both in the perilesional cortex and ipsilateral thalamus or down-regulated both in the perilesional cortex and ipsilateral thalamus are bold fonts

	Perilesional cortex					Ipsilateral thalamus				
TF	Log2FC	FDR	Altered targets/all targets	Upregulated TF targets	Downregulated TF targets	Log2FC	FDR	Altered targets/all targets	Upregulated TF targets	Downregulated TF targets
*Cebpd*	1.835	3.78E-034	1/1	**IGF1**	–	1.261	7.47E-005	1/1	**IGF1**	–
*Etv4*	−1.105	2.03E-008	0/1	–	–	−0.697	0.00523964	0/1	–	–
*Myb*	1.350	0.0042262	0/3	–	–	ns	ns	–	–	–
*Pax6*	1.186	5.97E-022	91/300	**ABCA1,** ABL1, BMP6, **BTK,** CAV2, CDKL1, DAB2, **DAPP1, ERBB2IP,** FGFR1, **FGR,** FOXO1, FYB, **GAB1, IL21R,** ITGA8, **ITGB1,** MAP3K1, MET, MICAL1, MTSS1, NEDD1, NIM1, PRKD1, PTPRZ1, ROR2, SKAP2, STK10, SYK, **TGFA,** TICAM2, UBE2U	AKT3, **ARHGAP32,** ATF2, BIRC6, BMPR2, **CAMK1D, CHN1,** CHRNA7, CNKSR2, CTNND2,DAB, DMXL2, EFNA5, EPHA7, EPHB2, ERBB4, ESRRG, GABRB3, GRB2,GRIA1, GRIA4, GRIK3, GRIN2A, ITPR1, MAGI2, MAP3K13, **MAPK10,** MAPK8IP3, MEF2C, MRAS, **NEDD4L,** NLK, NRD1, NRG1, NRG3, NUAK1, PAK7, PAM, PCSK5, PDE4D, PLCB1, PRKCA, **PRKCB,** PTPRG, PTPRJ, PTPRK, PTPRT, RAB14, **RAP1GDS1,** RGS7, ROCK2, RPS6KC1, RPTOR, SH3GL2, SNRK, SORBS2, TRIM33, TTBK2, ZFYVE9	0.779	8.41E-5	33/300	**ABCA1,** ATF1, **BTK, DAPP1,** EPHB1, **ERBB2IP,** ERBB4, FGF2, **FGR, GAB1,** GRB14, **IL21R,** INPP5D, ITGAM **ITGB1,** PKP4, PRKCA, TCF4, **TGFA**	**ARHGAP32, CAMK1D, CHN1,** DAPK1, GRM1, MAP3K14, **MAPK10, NEDD4L, NRG2, PRKCB, RAP1GDS1,** SRC, STRADB, TRIO
*Spi1*	1.021	2.88E-011	1/3	LSP1	–	1.310	8.57E-006	1/3	**LSP1**	–
*Tp73*	1.328	0.01004339	15/54	TGFA, PTPN22, CDK1, PDGFC, NOTCH1	BDNF, RGS7, **ERBB4,** SNCA, PRKCE, PTPRJ, NEDD4L, KPNA1, APBB2, STAM, MYO3B	ns	ns	6/55	MYLK, NEDD4L	RRAD, TGFA, **ERBB4,** PCNA

*Abbreviations*: *FDR* false discovery rate, *ns* non-significant, *TF* transcription factor

#### Validation of transcription factor expression in the perilesional cortex after TBI

In the perilesional cortex, validation confirmed a significant increase in gene expression of ***Cebpd*** (*p* = 0.0003996), ***Pax6*** (*p* = 0.004795), ***Spi1*** (*p* = 0.007592), and ***Tp73*** (*p* = 0.0007992) (Table 4) at 3 months after TBI.

**Table 4:** Gene expression validation of genes encoding transcription factors using ddPCR in the rat perilesional cortex, thalamus and hippocampus at three months after TBI

	*Cebpd*	*Pax6*	*Spi1*	*Tp73*
*Perilesional cortex*				
Fold change	**2.58**	**1.64**	**1.75**	**6.67**
p-value	**0.0003996**	**0.004795**	**0.007592**	**0.0007992**
*Thalamus*				
Fold change	1.55	1.13	1.65	0.55
p-value	0.2721	0.4559	0.06633	0.3884
*Hippocampus*				
Fold change	0.74	0.91	1.00	0.79
p-value	0.1375	0.7308	0.6282	0.6282

***Statistical significances:*** Fold change and corresponding *p*-value (< 0.05) are shown in bolded font

#### Transcription regulatory network of the ipsilateral thalamus

TRN analysis of the ipsilateral thalamus revealed upregulation of ***Pax6, Cebpd,*** and ***Spi1***, and downregulation of the ***Etv4*** transcription factor at 3 months post-TBI (Table 3). ***Pax6*** had 19 targets that were upregulated and 14 targets that were downregulated. ***Igf1***, the only target of ***Cebpd,*** was upregulated. ***Spi1*** had one upregulated target, ***Lsp1***. ***Etv4*** showed no alteration in gene expression.

#### Validation of transcription factor expression in the ipsilateral thalamus and hippocampus after TBI

In the ipsilateral thalamus, validation of gene expression indicated a trend toward increased expression of ***Spi1*** (*p* = 0.06633), but not ***Cebpd*** (*p* = 0.2721), ***Pax6*** (*P* = 0.4559), or ***Tp73*** (*p* = 0.3884) (Table 4) at 3 months after TBI. In the ipsilateral hippocampus, gene expression was unchanged.

### LINCS analysis revealed 118 candidate pharmacotherapies that can regulate transcription factors

#### Pharmacotherapies regulating transcription factor gene expression

The LINCS database analysis revealed 118 pharmacotherapies that can modify the gene expression of the top four transcription factors (***Pax6, Tp73, Cebpd,*** and ***Spi1)*** (Table 5). Expression of ***Cebpd*** was upregulated by 92 compounds and downregulated by two compounds. ***Pax6*** was upregulated by eight compounds and downregulated by six compounds. ***Spi1*** was upregulated by two compounds and downregulated by three compounds. ***Tp73*** was upregulated by five compounds. Interestingly, none of the compounds regulated more than one transcription factor.

**Table 5:** LINCS database analysis identified compounds that up-regulate or down-regulate the gene expression of *Cebpd*, *Pax6*, *Spi1* and *Tp73* transcription factors. Identification of compounds regulating the expression of transcription factors were carried out by retrieving compound-induced transcription profiles in terminally differentiated neurons, terminally differentiated neurons treated with KCl, and iPS-derived neural progenitor cells

TF	Upregulating compounds	Downregulating compounds
*Cebpd*	aminobenztropine (NEU), BG-1002 (NEU.KCL), BG-1011 (NPC), BIIB021 (NPC), BRD-A06779035 (NPC), BRD-A70591769 (NEU), BRD-A75769921 (NEU.KCL), BRD-A92334183 (NEU), BRD-K01608965 (NPC), BRD-K07381195 (NPC), BRD-K12683703 (NPC), BRD-K15050703 (NPC), BRD-K15935695 (NPC), BRD-K16934333 (NPC), BRD-K20126873 (NPC), BRD-K21374126 (NPC), BRD-K23986500 (NPC), BRD-K24798550 (NPC), BRD-K25164076 (NPC), BRD-K25990552 (NEU), BRD-K28934562 (NPC), BRD-K30229575 (NPC), BRD-K32885145 (NPC), BRD-K36269259 (NEU), BRD-K36313546 (NPC), BRD-K36591038 (NPC), BRD-K36796217 (NPC), BRD-K39597586 (NPC), BRD-K40300908 (NPC), BRD-K41871066 (NPC), BRD-K43631199 (NPC), BRD-K44540157 (NPC), BRD-K49111930 (NPC), BRD-K54331210 (NPC), BRD-K55536701 (NPC), BRD-K57166447 (NPC), BRD-K58808184 (NPC), BRD-K59253994 (NPC), BRD-K62970326 (NPC), BRD-K63494246 (NEU), BRD-K64523453 (NPC), BRD-K65148580 (NPC), BRD-K65657366 (NPC), BRD-K72354054 (NPC), BRD-K73008154 (NPC), BRD-K77888550 (NPC), BRD-K78133682 (NPC), BRD-K79947405 (NPC), BRD-K80062189 (NPC), BRD-K80138901 (NPC), BRD-K80400482 (NEU), BRD-K85133207 (NPC), BRD-K86110682 (NPC), BRD-K91844626 (NEU), BRD-K93158953 (NPC), BRD-K99718824 (NPC), cabergoline (NEU), chlorpromazine (NEU), deoxycholic-acid (NEU), econazole (NPC), farnesylthioacetic-acid (NPC), fluspirilene (NEU), geldanamycin (NPC), GSK-461364 (NPC), GW-3965 (NPC), GW-441756 (NPC), IQ1 (NEU), IQ1 (NPC), isoflupredone (NPC), ITSA-1 (NPC), IWP-2 (NEU), LY-255283 (NPC), LY-294002 (NPC), menadione (NPC), NVP-BEZ235 (NPC), PD-173074 (NPC), PI-828 (NPC), quercetin (NPC), R-96544 (NPC), scoulerine (NPC), serotonin (NPC), spermidine (NEU), SR-142948 (NEU), ST-023431 (NPC), ST-056792 (NPC), suberoyl-bis-hydroxamic-acid (NEU), tamoxifen (NPC), tozasertib (NPC), tranylcypromine (NEU.KCL), triacetylresveratrol (NPC), trichostatin-a (NEU), trifluridine (NPC), vorinostat (NPC)	BRD-K89824424 (NPC), O-1918 (NPC)
*Pax6*	acetyl-farnesyl-cysteine (NEU.KCL), BRD-K02409808 (NEU), BRD-K24656059 (NPC), BRD-K45842176 (NPC), clofibric-acid (NPC), rolipram (NPC), SKF-96365 (NPC), thioproperazine (NPC)	apicidin (NEU.KCL), BG-1016 (NPC), BRD-K37650321 (NEU), chrysamine-g (NEU), proadifen (NPC), XMD-1150 (NEU)
*Spi1*	BRD-K39172790 (NEU), timosaponin (NEU)	AS-703026 (NPC), genistein (NPC), U-0126 (NPC)
*Tp73*	BRD-K16827616 (NPC), BRD-K78133682 (NPC), RG-14620 (NPC), trimipramine (NEU), wortmannin (NEU)	–

*Abbreviations*: *NEU* terminally differentiated neurons, *NEU.KCL* terminally differentiated neurons treated with, *NPC* iPS-derived neural progenitor cells, *TF* transcription factor

## Discussion

In the present study, we evaluated whether TBI induces long-lasting transcriptomics regulation that is under the control of DNA-methylation. Bioinformatics analysis indicated that transcription factors rather than DNA methylation regulate gene expression at 3 months after TBI. Further, LINCS analysis revealed that several drugs already in clinical use modulate the expression of the identified key regulatory transcription factors ***Cebpd***, ***Pax6***, ***Spi1,*** and ***Tp73***.

### DNA methylation is not a major regulator of chronically altered post-TBI gene expression in our experimental model

Our previous study indicated regulated expression of approximately 5000 genes in the perilesional cortex and 1900 in the thalamus at 3 months after lateral FPI-induced TBI. in particular, we found a positive enrichment of inflammation-related genes and downregulation of ion channel-related genes [69]. The mechanisms that regulate chronic gene expression, however, remained unclear. Our transcriptomic data suggest that gene expression alterations could not be explained only by changes in the local cell populations [69]. One potential major regulator could be DNA-methylation, which regulates gene expression in several brain diseases, including Alzheimer’s’ disease [19], Parkinson’s disease [51], amyotrophic lateral sclerosis [32], epilepsy [127], and TBI [39, 83, 102, 106]. To explore the significance of DNA methylation as a regulator of chronically detected transcriptomics changes, we carried out genome-wide MBD-seq and RNA-seq from the perilesional cortex, ipsilateral thalamus, and ipsilateral hippocampus at 3 months post-TBI.

Regulated methylation was associated with altered gene expression only in the perilesional cortex. GSEA analysis revealed that the most enriched DNA methylation patterns in the perilesional cortex after TBI were in the gene promoter area. Specifically, MBD-seq indicated altered methylation in promoters of the ***Lrp1b***, ***Gpr12, Wrd26,*** and ***Ppid*** genes. Pyrosequencing, however, did not confirm the sequencing data. This could relate to a low, although consistent, read count per methylation site in the promoter region (< 200) in the MBD-seq. Our negative findings are in agreement with previous studies in fluid-percussion injury, controlled cortical injury, weight-drop, and blast-induced TBI models, which reported no changes in the promoter methylation of ***Lrp1b***, ***Gpr12, Wrd26*** and ***Ppid*** genes in cortical, hippocampal, or amygdaloid tissue sampled 3 days to 8 months post-injury [39, 83, 102, 106]. We were not able to reproduce the altered methylation in the rat cortex at 8 months after blast TBI [39], in the rat amygdala at 48 h to 30 d after weight-drop–induced TBI [102], in the rat hippocampus at 3 and 14 d after controlled cortical impact [106], in the rat hippocampus at 7 d after fluid-percussion injury [83], or in the rat hippocampus 3 months after lateral fluid-percussion injury [20]. This is likely related to the different injury types and post-injury delays between the present and previous experimental studies.

### TRN analysis revealed transcription factors *Cebpd*, *Pax6*, *Spi1* and *Tp73* as regulators of chronically altered post-TBI gene expression

To identify the master switch that regulates the massively altered gene expression at 3 months post-TBI, we next investigated the possible contribution of transcription factors by integrating transcriptomics data into the Signalink 2.0 database. Laboratory validation of top hits using ddPCR confirmed the predicted mRNA upregulation of four transcription factors, ***Pax6***, ***Tp73, Cebpd***, and ***Spi1,*** in the perilesional cortex.

***Pax6*** showed a 6.7-fold upregulation, and almost 30% (91 or 300) of its target genes were regulated in the perilesional cortex. The function of ***Pax6*** in the cerebral cortex is unknown. In the hippocampus, ***Pax6*** controls the differentiation and migration of neuronal progenitor cells (NPC) [37], which show regenerative potential after ischemic injury [88]. Moreover, overexpression of ***Pax6*** improved the cellular viability of SH-SY5Y cells exposed to neurotoxin [119]. These studies suggest that upregulation of ***Pax6*** after TBI could play a reparative role.

***Tp73*** showed a 1.6-fold upregulation, and approximately 30% (15 of 54) of its target genes were regulated in the perilesional cortex after TBI. The Tp73 gene has two promoters, producing two protein isoforms with different functions [86]. The ***ΔNp73*** isoform is anti-apoptotic during development of the mouse superior cervical ganglion neurons [95]. Furthermore, ***ΔNp73*** is vital for long-term survival of mouse superior cervical ganglion and cortical neurons [66, 94]. The ***TAp73*** isoform, however, induces apoptosis in SAOS-2 and medulloblastoma cell cultures [11, 50]. Further studies are needed to explore the functional consequences of Tp73 upregulation after TBI.

***Cebpd***, also known as NF-IL6β, regulates immune and inflammatory responses [57, 101]. ***Cebpd*** showed a 2.6-fold upregulation, and its predicted target gene, IGF-1, was also upregulated in the perilesional cortex at 3 months post-TBI. There are some reports of increased expression of ***Cebpd*** in the cortex already at 2 h to 7 d after injury in various experimental models of TBI, including a controlled cortical impact mouse model [48, 105, 128] and a weight-drop rat model [16, 123]. These studies propose a wide time window for the post-injury regulation of ***Cebpd*** and its target IGF-1. It remains a testable hypothesis that chronic upregulation of ***Cebpd*** and *IGF-1* genes relates to the control of chronic inflammation after TBI, as recently suggested in favorable proof-of-concept preclinical studies in injury models [75, 96, 110, 120].

***Spi1*** showed a 1.8-fold upregulation at 3 months post-TBI. ***Spi1*** is expressed in microglia [126], which become activated after TBI [13, 61, 124]. ***Spi1*** encodes PU.1, which appears vital for microglial survival [111]. Our TRN network analysis revealed three gene targets for ***Spi1***, and of those, ***Lsp1*** was upregulated in the perilesional cortex and ipsilateral thalamus at 3 months post-TBI. ***Lsp1*** was reported to be upregulated in the rat cerebral cortex at 24 h after controlled cortical impact-induced TBI [128]. ***Spi1*** regulates monocyte and macrophage differentiation [100], and has a crucial role in the normal development of T cells, B cells, neutrophils, and macrophages [82], which are important players in the post-TBI systemic inflammatory response [110].

In summary, our findings indicate that four transcription factors, ***Pax6, Tp73, Cepbd***, and ***Spi1,*** serve as major chronic post-TBI transcriptomics regulators, and are thus potential targets for treatments.

### LINCS analysis revealed transcription factor-targeting antidepressants and anti-cancer drugs as novel treatment candidates for TBI

Next, we performed a LINCS analysis to identify compounds that modulate the gene expression of ***Pax6, Tp73, Cepbd****,* or ***Spi1***. The largest number of compounds identified targeted ***Cebpd***. Most of the compounds upregulating ***Cebpd*** were bioactive (starting with BRD) without any known therapeutic actions. The analysis, however, also identified antidepressants and anti-cancer drugs that are already used in the clinic. **Tranylcypromine**, an antidepressant and monoamine oxidase inhibitor (MAO-I) [29], is a promising therapy as MAO-Is have neuroprotective effects in mice with TBI [44]. Tranylcypromine also alleviates neurodegeneration and inflammation by inhibiting prostacyclin and arachidonic acid release in calf primary endothelial cells [34, 43]. LINCS analysis also revealed **fluspirilene and chlorpromazine** as upregulators of ***Cepbd****.* Duotherapy with **chlorpromazine** and promethazine was demonstrated to be neuroprotective when assessed at 24 h after brain ischemia in rats [35]. **Chlorpromazine** suppressed neuronal apoptosis in the rat parietal cortex and the CA1 subfield of the hippocampus when assessed at 24 h after ethanol-induced apoptosis [131]. **Chlorpromazine** also reduced the cerebral infarct size when assessed at 24 h post-ischemia in rats [68].

In addition to compounds used in psychiatry, LINCS analysis revealed an anti-cancer drug, **vorinostat**, a histone deacetylase inhibitor (HDAC1–3 and 6) [77] as an upregulator of ***Cepbd***. **Vorinostat** attenuated neurodegeneration and improved neurological outcome when assessed at 24 h after stroke in rats [117]. Interestingly, valproate, another HDAC inhibitor (HDAC1–3 and 8) [6], is neuroprotective and anti-inflammatory in rodent models of TBI and ischemia [18, 55, 132]. **Tamoxifen** was another upregulator of ***Cepbd*** identified by the LINCS analysis. Tamoxifen is a selective estrogen receptor modulator [107] used to treat breast cancer [33]. Tamoxifen reduced the cerebral infarct volume and neuronal apoptosis when assessed at 72 h after fluid-percussion injury in rats [121]. LINCS analysis also revealed two compounds that downregulated ***Cebpd***, O-1918 and BRD-K89824424***.*** Information available from **O-1918** indicates that it is a cannabidiol analog, acting as a selective antagonist of abnormal cannabidiol at the non-CB_1_/CB_2_ endothelial receptor [89, 135]. Interestingly, another cannabinoid receptor antagonist, AM630, counteracted the recovery-enhancing effects of leptin [71]. Moreover, SR144528, a cannabinoid receptor antagonist increased TNFα gene expression 24 h after mouse controlled cortical impact (CCI), suggesting that it enhances the inflammatory response [2]. Taken together, these studies suggest that upregulation of ***Cebpd*** favorably modifies the post-TBI outcome.

LINCS analysis revealed **SKF-96365, thioproperazine,** and **rolipram** as upregulators of ***Pax6***. **SKF-96365** is an inhibitor of receptor-mediated calcium entry [84]. In an in vitro model of bovine brain microvessel endothelial cells, SKF-96365 decreased blood-brain barrier permeability [1], a major pathology in TBI. **Thioproperazin** is a neuroleptic that increases dopamine release [12]. Interestingly, dopamine release was decreased at 1 week after TBI in the rat CCI model [109], and an increase in dopamine level by methylphenidate improved spatial memory based on a shorter Morris water-maze latency at 14 d after rat CCI [58]. **Rolipram**, an antidepressant, MAO-I, and phosphodiesterase (PDE) IV inhibitor, also upregulates Pax6 gene expression. It suppresses cytokine production in human and rat T cells [114]. Moreover, **rolipram** inhibits neuronal damage in gerbil CA1 hippocampus 7 d after stroke [53]. **Rolipram** also reduced infarct size, improved neurological outcome, increased anti-inflammatory cytokines, and decreased pro-inflammatory cytokines at 24 h after mouse focal cerebral ischemia [62]. LINCS analysis revealed that ***Pax6*** was downregulated by **proadifen**, a cytochrome P-450 inhibitor [10], and by **apicidin**, a histone deacetylase inhibitor [41]. Interestingly, **apicidin** induced apoptosis in MCF-7 cells through cell cycle regulatory proteins [45] and reversed nitric oxide and inducible nitric oxide synthase expression induced by dexamethasone and RU24858 in a mouse macrophage cell culture [40]. Taken together, upregulation of ***Pax6*** gene expression appears to be a target for favorable modulation of the post-TBI outcome by reversing the reduced dopamine release and reducing neuroinflammation via cytokine release, as suggested by studies of **thioproperazine** and **rolipram**. Moreover, both ***Cepbd*** and ***Pax6*** are upregulated by compounds with an MAO-I mechanism, and are predicted to have favorable effects.

LINCS analysis revealed two compounds that upregulate ***Spi1.*** From these, **timosaponin AIII** is a candidate anti-cancer drug [52, 118]. It reverses scopolamine-induced memory impairment in mice [67]. LINCS analysis revealed three compounds that downregulated ***Spi1***. **AS-703026** (also known as pimasertib) and **U0126** are MEK1/2 inhibitors [30, 56]. **U0126** has favorable effects on recovery in various in vivo brain injury models. For example, it reduced infarct size when assessed at 24 h after middle cerebral artery occlusion in rats [27], lesion size when analyzed at 7 d after in mice injured with controlled cortical impact [87], and microglial activation in the ischemia model of spinal cord injury in rats [73]. **Genistein**, a phytoestrogen with a broad spectrum of pharmacological properties, inhibits protein tyrosine kinases and topoisomerase II, and exhibits estrogen-like activity [23, 93]. **Genistein** showed neuroprotective effects when assessed at 48 h after weight-drop–induced TBI [113] and at 24 h after focal cerebral ischemia in rats [4]. Whether downregulators of ***Spi1*** will have favorable effects on more chronic post-TBI outcome remains to be investigated.

LINCS analysis revealed three compounds with some prior information of biological effects, all of which upregulated ***Tp73.*** **Wortmannin,** a radiosensitizer, is a phosphoinositide 3-kinase inhibitor [5] that also inhibits mTOR in vitro*,* a pathway involved in post-TBI recovery and epileptogenesis in several post-injury animal models [8, 92]. **Trimipramine** is a tricyclic antidepressant [103] that reduced interferon-γ production, suppressed T-cell proliferation, and increased interleukin-12 production in concanavalin A-stimulated human whole blood cultures [21]. The effects of **trimipramine** on brain injury, however, are poorly described. **RG-14620** is a protein tyrosine kinase inhibitor with antiproliferative effects [134]. Compounds that upregulate ***Tp73*** expression are interesting candidates for further studies. For example, trimipramine is already used in the clinic and could be repurposed to improve outcome after TBI.

## Conclusions

This is the first analysis of chronic regulation of gene expression after TBI, demonstrating that chronic post-TBI transcriptional regulation is more under the control of transcription factors than DNA methylation. In particular, four upregulated transcription factors ***Pax6, Tp73****,* ***Cebpd****,* and ***Spi1,*** appeared as potent regulators of chronic post-TBI gene expression. They regulate the molecular networks contributing to post-injury secondary damage, including apoptosis and inflammation, strengthening the feasibility of therapeutically targeting these molecular networks even after the acute post-TBI period. To complement hypothesis-driven therapeutic approaches, our systems-biology driven unbiased LINCS database analysis revealed several novel treatment candidates. In particular, our data together with a literature search of effects in in vitro and in vivo models of brain injury revealed that antidepressant/neuroleptics such as trimipramine, rolipram, fluspirilene, and chlorpromazine, as well as the anti-cancer therapies pimasertib, tamoxifen, and vorinostat are candidates for further testing to favorably modulate regulated transcriptomics networks and post-TBI outcome.

## Additional files

Additional file 1: Pyrosequencing assays. (DOCX 16 kb)

Additional file 2: Gene expression of astrocyte, microglia and neuronal markers in the perilesional cortex and ipsilateral thalamus in RNA-seq dataset. (DOCX 16 kb)

Additional file 3: Dot plots and correlations of the read counts of transcription factors with the read counts of neuronal, microglial and astroglial markers. (DOCX 55 kb)

## Abbreviations

CCI: Controlled cortical impact
ddPCR: Digital droplet polymerase chain reaction
FDR: False discovery rate
FPI: Fluid-percussion injury
GSEA: Gene Set Enrichment Analysis
HDAC: Histone deacetylase inhibitor
MAO-I: Monoamine oxidase inhibitor
MBD-seq: Methyl-binding domain sequencing
NEU: Terminally differentiated neurons
NEU.KCL: Terminally differentiated neurons treated with KCl
NPC: iPS-derived neural progenitor cells
RIN: RNA integrity number
RNA-seq: RNA-sequencing
STAR: Spliced Transcripts Alignment to a Reference
TBI: Traumatic brain injury
TRN: Transcription regulatory network
