# Supplementary material for: Transcription factors Tp73, Cebpd, Pax6, and Spi1 rather than DNA methylation regulate chronic transcriptomics changes after experimental traumatic brain injury
Source: Acta Neuropathol Commun. 2018 Feb 27;6:17. doi: 10.1186/s40478-018-0519-z (PMC5828078; doi:10.1186/s40478-018-0519-z)
Supplement: Supplementary file 1 — Pyrosequencing assays. (DOCX 16 kb) [file 40478_2018_519_MOESM1_ESM.docx]

**Transcription factors Tp73, Cebpd, Pax6, and Spi1 rather than DNA methylation regulate chronic transcriptomics changes after experimental traumatic brain injury**

Anssi Lipponen, Assam El-Osta, Antony Kaspi, Mark Ziemann, Ishant Khurana, Harikrishnan KN, Vicente Navarro-Ferrandis, Noora Puhakka, Jussi Paananen, Asla Pitkänen

Additional file 1

Pyrosequencing assays and CpG-sites which of methylation status were assessed. CpG-sites of which bolded and underlined.

*Wdr26*

>5’-3’ 13 dna:chromosome chromosome:Rnor_5.0:13:104530432:104530766:1

ggcttttttttttttcttttttcttttttttccggagctggggaccgaacccagggccttgcacttgctaggcaagcgctctaccactgagctaaatccccaaccctaagcggcttttttgagtggagaaacacaGCtGCggagaGCtgaGCtgtatgttattcaatgGCgaatggtccacaggaccctagtcagttacatgtagttgtgtggtaG**Cg**tgtaggacctggtgtgttcagatattttccctgacgtttaggaaattcttggtgactttgggagggaaaacaacaataataacaacaaaaaacaaacaaacaggcaaacctcaacag

| Primer | Sequence |
| --- | --- |
| Forward | GGTTTTTTTGAGTGGAGAAATATAGT |
| Reverse | [Btn]CCCAAAATCACCAAAAATTTCC |
| Sequence | ATGTAGTTGTGTGGTAG |

*Lrp1b*

>5’-3’ Lrp1b 3 dna:chromosome chromosome:Rnor_5.0:3:31168391:31168788:1

aagtgtttttggatttgttttcatagtttgcagtatctactcacccacactgatgcagctttccccatcagtatccaacgcccagccttcatagcaggagcacttgaccatgtgtttgtgctgttcgcacacttggctacacttcaggtgggaggagcagtagtctacgatttcacaggttctattgtccttcttgaGCtgatgtccttctggGCaggaacagacaattccttttccaggtacaaCGgaacagtggttGCtacaGCCtCCGttgttcagagaacattcatctgtcaagagacaaa**cg**ttcttagtcagatagtttatctgagttcatgctactcattgattgaattctctaagtctagaattacagtcagagattttatgacccaata

| Primer | Sequence |
| --- | --- |
| Forward | TGGGTTATAAAATTTTTGATTGTAAT |
| Reverse | [Btn]AACAAACAATTCCTTTTCCAAATAC |
| Sequence | AGATAAATTATTTGATTAAGAA |

*Ppid*

>Ppid 2 dna:chromosome chromosome:Rnor_5.0:2:197689347:197689713:1

ctggcctggaactcacgtggatcctcctgcctctacctcctcaggactgaataagcacattcagctttaagacgtatattttacaccaagctcatttggaaaaaaaaaataataatagcgtgtcctggggttggggatttagctcagcggtaaaGCccttGCctagggaGCGCaagGCcctgggtt**CG**gaccccaGCtCCGaaaaaaaaaaaaaatagcgtgtccagttttaacagaagtcaaaatcctctcaatatcggcgcatttggagcctttgctgtagactgggtcactaacaacgtgatggccaaatgacaataacattgtaatagtaacaggacagttgttattaggagccacaagttta

| Primer | Sequence |
| --- | --- |
| Forward | GTGTTTTGGGGTTGGGGATTTA |
| Reverse | [Btn]CCCAATCTACAACAAAAACTCCAAATAC |
| Sequence | GAGAGTAAGGTTTTGGGT |

*Gpr12*

>5’-3’ Gpr12 12 dna:chromosome chromosome:Rnor_5.0:12:12357191:12357630:1

gagtcactgcgtttgcccatatggcagggggctcggtggttcaagactatctttgaattatcagttaaggtttccatccctgtaacacaacaccatggccacggcgactcacaggagaaagcatttaaccagggttacagttccagatggtcagagtc**CG**tGCtcagGCatctcagacaGCctcaGCtctagG**Cg**tgaGCctacttaGCtcctcctttCGaatcttcttgggcctcagcacaagacccccagcacacctcagcacacctctgccttcgtccagcacttcgaactgagttctctctccttatgacccagacaagccgcagagagggaactccaggcttgaggaagagctcaggggtaggacacactcccatcagagcaagactgtggattcagattcgatccttggctctgagggggaaacctgggcaaac

| Primer | Sequence |
| --- | --- |
| Forward | [btn]GATTAGAAAGGAGGAGTTAAGTAGGT |
| Reverse | ATCCCTATAACACAACACCATAAC |
| Sequence | ACAATTCCAAATAATCAAAATCC |
