# Supplementary material for: Transcription factors Tp73, Cebpd, Pax6, and Spi1 rather than DNA methylation regulate chronic transcriptomics changes after experimental traumatic brain injury
Source: Acta Neuropathol Commun. 2018 Feb 27;6:17. doi: 10.1186/s40478-018-0519-z (PMC5828078; doi:10.1186/s40478-018-0519-z)
Supplement: Supplementary file 2 — Gene expression of astrocyte, microglia and neuronal markers in the perilesional cortex and ipsilateral thalamus in RNA-seq dataset. (DOCX 16 kb) [file 40478_2018_519_MOESM2_ESM.docx]

| Gene expression of astrocyte, microglia and neuronal markers in the perilesional cortex and ipsilateral thalamus in RNA-seq dataset at 3 months post-TBI (Lipponen *et al* 2016). Note an increase in the expression of glial and a decrease in neuronal markers. | | | | |
| --- | --- | --- | --- | --- |
|  | **Cortex** | | **Thalamus** | |
|  | **Log2FC** | **FDR** | **Log2FC** | **FDR** |
| **Astrocyte markers** | | | | |
| *Gfap* (GFAP) | 2.6 | 2,96E-70 | 2.05 | 2.87E-10 |
| *Aldh1l1* | 0.66 | 1,77E-08 | 0.29 | 0.289559908 |
| **Microglia markers** | | | | |
| *Aif1* (Iba1) | 0.61 | 0,000165347 | 0.45 | 0.081521484 |
| *Cd68* | 2.3 | 4,69E-26 | 2.4 | 6.32E-07 |
| **Neuronal markers** | | | | |
| *Rbfox3* (NeuN) | -0.38 | 0,039147077 | -0.23 | 0.307629881 |
| *Tubb3* (TuJ) | -0.46 | 0,000721864 | -0.48 | 0.001720187 |

**Transcription factors Tp73, Cebpd, Pax6, and Spi1 rather than DNA methylation regulate chronic transcriptomics changes after experimental traumatic brain injury**

Anssi Lipponen, Assam El-Osta, Antony Kaspi, Mark Ziemann, Ishant Khurana, Harikrishnan KN, Vicente Navarro-Ferrandis, Noora Puhakka, Jussi Paananen, Asla Pitkänen

Additional file 2

Reference

Lipponen A, Paananen J, Puhakka N, Pitkänen A. Analysis of Post-Traumatic Brain Injury Gene Expression Signature Reveals Tubulins, Nfe2l2, Nfkb, Cd44, and S100a4 as Treatment Targets. Sci. Rep. 2016;6:31570.
