# Supplementary material for: Transcription factors Tp73, Cebpd, Pax6, and Spi1 rather than DNA methylation regulate chronic transcriptomics changes after experimental traumatic brain injury
Source: Acta Neuropathol Commun. 2018 Feb 27;6:17. doi: 10.1186/s40478-018-0519-z (PMC5828078; doi:10.1186/s40478-018-0519-z)
Supplement: Supplementary file 3 — Dot plots and correlations of the read counts of transcription factors with the read counts of neuronal, microglial and astroglial markers. (DOCX 55 kb) [file 40478_2018_519_MOESM3_ESM.docx]

**Transcription factors Tp73, Cebpd, Pax6, and Spi1 rather than DNA methylation regulate chronic transcriptomics changes after experimental traumatic brain injury**

Anssi Lipponen, Assam El-Osta, Antony Kaspi, Mark Ziemann, Ishant Khurana, Harikrishnan KN, Vicente Navarro-Ferrandis, Noora Puhakka, Jussi Paananen, Asla Pitkänen

Additional file 3

Dot plots represent the correlation between RNA-seq raw read counts of transcription factors **(A)** *Pax6* and **(B)** *Tp73* with a neuronal marker *Rbfox3*, microglial marker *Aif1*, and astroglial marker *Gfap* (Lipponen *et al*. 2016). Note that the expression of transcription factors correlates between *Pax6* and the microglial marker as well as between *Pax6* and *Tp73* and the astroglial marker in the same animals at 3 months post-TBI.

r_s_=0.806

p=0.005

**B**

**A**

r_s_=-0.212

p=0.556

r_s_=-0.030

p=0.934

r_s_=0.721

p=0.019

r_s_=0.915

p=0.000

r_s_=0.721

p=0.019

Reference

Lipponen A, Paananen J, Puhakka N, Pitkänen A. Analysis of Post-Traumatic Brain Injury Gene Expression Signature Reveals Tubulins, Nfe2l2, Nfkb, Cd44, and S100a4 as Treatment Targets. Sci. Rep. 2016;6:31570.
